# A review on mitochondrial restorative mechanism of antioxidants in Alzheimer’s disease and other neurological conditions

**DOI:** 10.3389/fphar.2015.00206

**Published:** 2015-09-24

**Authors:** Anil Kumar, Arti Singh

**Affiliations:** Pharmacology Division, University Institute of Pharmaceutical Sciences, UGC Centre of Advanced Study, Panjab University, Chandigarh, India

**Keywords:** Alzheimer’s disease, mitochondria, mitochondrial dysfunction, oxidative stress, coenzyme Q10

## Abstract

Neurodegenerative diseases are intricate in nature because of the involvement of the multiple pathophysiological events including mitochondrial dysfunction, neuroinflammation and oxidative stress. Alzheimer’s disease (AD) is a neurodegenerative disease explained by extracellular amyloid β deposits, intracellular neurofibrillary tangles and mitochondrial dysfunction. Increasing evidence has indicated that mitochondrial dysfunction displays significant role in the pathophysiological processes of AD. Mitochondrial dysfunction involves alterations in mitochondrial respiratory enzyme complex activities, oxidative stress, opening of permeability transition pore, and enhanced apoptosis. Various bioenergetics and antioxidants have been tried or under different investigational phase against AD and other neurodegenerative disorders (Parkinson’s disease, Huntington’s disease, and Amyotrophic lateral sclerosis) because of their complex and multiple site of action. These mitochondrial-targeting bioenergetics and antioxidant compounds such as coenzyme Q10, idebenone, creatine, mitoQ, mitovitE, MitoTEMPOL, latrepirdine, methylene blue, triterpenoids, SS peptides, curcumin, *Ginkgo biloba*, and omega-3 polyunsaturated fatty acids with potential efficacy in AD have been identified. Present review is intent to discuss mitochondrial restorative mechanisms of these bioenergetics and antioxidants as a potential alternative drug strategy for effective management of AD.

## Introduction

Alzheimer’s disease (AD) common incapacitating neurodegenerative disease, identified by the occurrence of senile plaques extracellularly and neurofibrillary tangles intracellularly (Alzheimer’s, 2015; Kumar et al., 2015). Globally AD is becoming epidemic because of new cases in every 7 s and more than 36.5 million individuals are affected worldwide (Prince et al., 2014; Alzheimer’s, 2015). Senile plaque consists of amyloid β (Aβ) peptides and tangles are made from tau protein. It was found that Aβ peptides are produced by the proteolytic segmentation of the protein known as amyloid precursor protein (APP). Other characteristic features of AD include progressive and neuronal synaptic loss, mitochondrial dysfunction, oxidative stress and inflammatory responses (Iqbal and Grundke-Iqbal, 2010; Swerdlow et al., 2014; Alzheimer’s, 2015). An increasing body of evidences indicates that Aβ enhances neuronal vulnerability to mitochondrial dysfunction via an impairment of electron transport chain (ETC) and oxidative stress (Davis et al., 1995; Reddy and Beal, 2008; Reddy et al., 2010; Calkins et al., 2011; Swerdlow et al., 2014).

Till date, rigorous efforts have been made to understand the complex pathophysiological mechanisms’ underlying AD and other neurodegenerative disorders including Parkinson’s disease (PD), Huntington’s disease (HD), and Amyotrophic lateral sclerosis (ALS) but still it remains ill-defined and poorly understood disease. There have been many hypotheses put forward to explain their complex pathophysiology such as inflammatory, mitochondrial dysfunction and oxidative stress hypotheses (Kumar and Singh, 2015). Out of these hypotheses, mitochondrial dysfunction and oxidative stress hypotheses are the most argued one (Davis et al., 1995; Swerdlow et al., 2014). As these two events are occurring at very early stages of neurodegenerative diseases so these can be one of the important and potential therapeutic targets in the current scenario (Swerdlow et al., 2014).

Mitochondria, the major organelles in neurons (Chaturvedi and Beal, 2013) and via oxidative phosphorylation (OXPHOS) or mitochondrial respiratory chain produce energy as adenosine triphosphate (ATP; Schapira, 2012; Zeviani et al., 2012). Other functions of mitochondria are the regulation of calcium homeostasis, generation of free radicals and apoptosis (Swerdlow, 2011). Strong evidence indicated that mitochondrial dysfunction involves alterations of mitochondrial respiratory chain enzymes, generation of reactive oxygen species (ROS), opening of mitochondrial permeability transition pore (mPTP), structural abnormalities of mitochondria, oxidative stress and apoptosis (Hauptmann et al., 2006; Lin and Beal, 2006; Eckert and Müller, 2014). And these mitochondrial abnormalities are known to occur early in AD before Aβ deposition and are closely related to Aβ- or tau- pathology (Swerdlow et al., 2010; Maruszak and Żekanowski, 2011).

Various compounds have been demonstrated to possess mitochondrial restoring and anti-oxidant properties such as coenzyme Q10 (CoQ 10), vitamin E, curcumin, *Gingko biloba*, melatonin and lipoic acid (Du and Yan, 2010). Therapeutic potentials of these compounds have been suggested to reduce Aβ peptides accumulations, restoring mitochondrial functions, transport and synaptic plasticity, protect mitochondria from Aβ toxicity, attenuate cognitive impairment in AD, inhibit dopaminergic neuronal loss in PD and showed neuroprotective role in other neurodegenerative disorders like ALS, HD (Hauptmann et al., 2006; Lin and Beal, 2006; Pieczenik and Neustadt, 2007; Moreira et al., 2010; Maruszak and Żekanowski, 2011).

One of the important tasks of this review is to discuss the growing evidences demonstrating the importance of mitochondria and related features in the pathogenesis of AD. Finally, we will discuss mitochondrial dysfunction as a potential drug target for AD management. The authors also projected various drug strategies targeting mitochondrial dysfunction and oxidative stress which may help in attenuation of AD, PD, HD, and ALS pathologies. Also, an attempt has been made to discuss various compounds targeting mitochondria and oxidative stress as a future approach with major focus on AD pathology.

## Mitochondrial Cascade Hypothesis

As discussed earlier, that the pathology of AD involves the extracellular aggregation of Aβ plaques and intracellular neurofibrillary tangles (Kumar and Singh, 2015). It was first suggested by Swerdlow in 2004 (Swerdlow and Khan, 2004) and according to this hypothesis; mitochondrial dysfunction is considered to be an early as well as a primary event in the pathophysiological cascade of AD (Swerdlow et al., 2010). Also it was proposed that genetic hereditary regulate mitochondrial functions and membrane strength, which changes with age and hence results in the development of AD related symptoms (Swerdlow and Khan, 2004; Swerdlow et al., 2010). This hypothesis assumed that autosomal dominant and sporadic AD are not etiologically same (Swerdlow et al., 2014). Mitochondrial dysfunction presents a connecting link between sporadic AD and autosomal dominant. In autosomal dominant AD, excessive Aβ accumulation slowly impairs mitochondrial functions which further initiate other AD related pathologies such as oxidative stress or neuroinflammation. In sporadic AD, age related occurrence of mitochondrial dysfunctions causes a variety of pathologies including oxidative stress and apoptosis (Hauptmann et al., 2006; Lin and Beal, 2006; Moreira et al., 2010; Witte et al., 2010).

## Mechanism of Mitochondrial Dysfunction in AD

**FIGURE 1:**
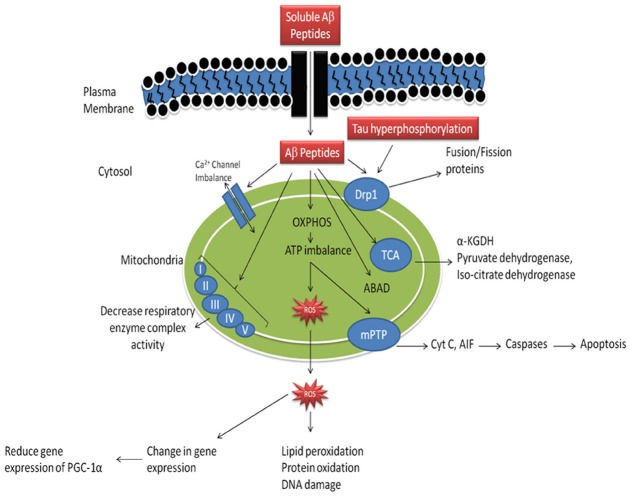
**Mechanism of mitochondrial dysfunction in Alzheimer’s disease.** (Aβ-amyloid β, OXPHOS- oxidative phosphorylation, ROS- reative oxygen species, mPTP- mitochondrial permeability transition pore, Cyt C- cytochrome C, Drp1- dynamin—related protein-1, ABAD- amyloid β binding alcohol dehydrogenase, α-KGDH- α-ketoglutarate dehydrogenase complex, PGC-1α- peroxisome proliferator activated receptor-γ-coactivator-1-α). Adopted and modified from Reddy and Beal (2008).

As reported earlier, in AD there is abnormal APP metabolism and an excessive Aβ accumulation (Kumar and Singh, 2015). It has been reported that Aβ peptides are present in the neuronal cells as well as in mitochondria (Swerdlow and Khan, 2004). When Aβ peptides accumulates in mitochondria it causes inhibition of mitochondrial respiratory enzyme complex-II and IV, causes decreased production of ATP and an increased production of ROS mitochondrial dysfunction in AD (Figure 1, Rhein et al., 2009; Swerdlow et al., 2010). Accumulation of Aβ peptides also known to reduce activity of enzyme of the tricarboxylic acid (TCA) cycle, α-ketoglutarate dehydrogenase (αKGD), pyruvate dehydrogenase and isocitrate dehydrogenase (Huang et al., 2003; Bubber et al., 2005). It is reported that, Aβ peptides interact with the Aβ binding site known as Aβ binding alcohol dehydrogenase (ABAD) which are present in the mitochondrial membranes and causes mitochondrial dysfunction (Lustbader et al., 2004), abnormal mitochondrial trafficking and decreased mitochondrial movement finally leads to synaptic degeneration (Calkins and Reddy, 2011). Further, Aβ peptide accumulation leads to dysfunctioning of mitochondrial Ca^2+^ channels, opening of mPTP and enhancement of cytochrome C (CytC) release (Calkins et al., 2011). Moreover, Aβ peptide accumulation inhibits protein import inside the mitochondria, which leads to mutation of mitochondrial DNA (mtDNA) and its damage (Lakatos et al., 2010). Mutations in APP also cause alterations of Ca^2+^ homeostasis leading to apoptosis (Khan et al., 2000). Accumulation of Aβ peptide and hyperphosphorylation of tau causes increased DRP-1nitrosylation which in turn causes abrupt mitochondrial fission and neurodegeneration (Manczak et al., 2011). Accumulation of soluble Aβ peptide and mutant APP impairs mitochondrial fusion and fission functions, abnormal mitochondrial movement, morphology and degradation of mitochondria (Manczak et al., 2011). Besides, it has also been reported that Aβ peptide accumulation causes abnormal expression of mitochondrial fission (Fis1) and fusion (mfn1/2 and OPA1) proteins which are involved in mitochondrial fission and fusion machinery (Manczak et al., 2011). These impaired dynamics causes decreased clearance of defective mitochondria which further enhanced neurodegeneration (Manczak et al., 2011). It has been studied that Aβ peptides induced hyperphosphorylation of tau causes inhibition of fission protein DRP1 which leads to abnormal mitochondrial elongation (Wang et al., 2008). In another study, Aβ peptides decrease proliferator-activated receptor-γ coactivator-1 α (PGC-1α) expressions, which leads to decreased mitochondrial biogenesis, mitochondrial DNA content (mtDNA) and increased neurodegeneration (McGill and Beal, 2006). Activation of PGC-1α causes increased non-amyloidogenic processing of APP, reduction in Aβ levels leading to increased survival of neuronal cells (McGill and Beal, 2006).

## Mitochondria in AD

Mitochondria are the major energy producing cell organelles and also known as the power house of the cell (Du and Yan, 2010). Studies have expanded the role of the mitochondrial genome, consisting of maternally-inherited multiple copies of semi-autonomous genome mtDNA along with thousands of nuclear DNA (nDNA)-encoded genes (Castellani et al., 2002; Selfridge et al., 2013). The mtDNA encodes major components of mitochondrial OXPHOS which includes specifically ETC (Castellani et al., 2002). On the other side, nDNA encodes the genes which are required for the assembly of mitochondria and its related structural elements (Castellani et al., 2002; Selfridge et al., 2013). Therefore, mitochondrial respiratory rate, oxidative stress and apoptosis, which constitute important byproduct of OXPHOS (Selfridge et al., 2013).

In 1995, Jane C. Chisholm and his team proposed “mitochondrial bottle neck hypothesis.” According to this hypothesis, mitochondria represent a unique target for therapeutic interventions for all forms of AD (Davis et al., 1995). Besides, mitochondrial functions are essential as well as their dysfunction are sufficient enough to cause neurodegenerative disorder, including AD and thus providing mechanistic bottleneck in neurodegenerative diseases. In AD, multiple heterogeneous natures cause the same pathological phenotype in mitochondria. This hypothesis also proposed that mitochondrial dysfunction is a common pathway in different neurodegenerative disorders (Davis et al., 1995).

It has been reported that several mitochondrial respiratory enzymes (pyruvate dehydrogenase complex, ketoglutarate dehydrogenase complex, and cytochrome oxidase) are altered in AD (Moreira et al., 2010). According to the mitochondrial cascade hypothesis, defect in cytochrome oxidase is considered to be central. Cytochrome oxidase is the major enzymes of the terminal end of mitochondrial ETC. It accepts electron from cytochrome c, which in turn receive it from the upstream part of ETC. Cytochrome oxidase transfer electron to oxygen to form H_2_O rather than ROS. And this enzyme is the common site for all cellular oxygen consumption (Swerdlow et al., 2014).

**FIGURE 2:**
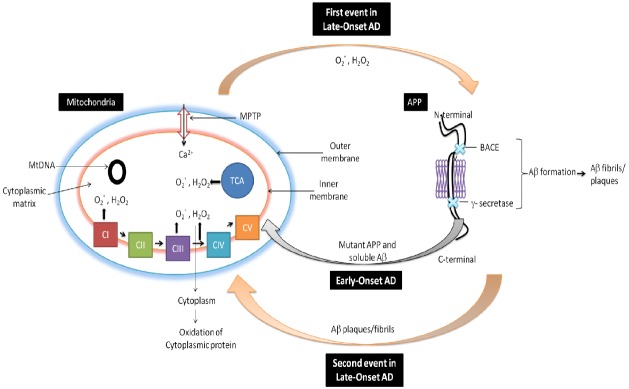
**Role of mitochondria in AD.** In early-onset AD, it is hypothesized that mitochondria leads to the generation of free radicals (${\text{O}}_{\text{2}}^{\cdot —}$, H_2_O_2_), which in turn decrease cytochrome oxidase activity and inhibit cellular ATP generation. Further in late-onset AD, free generation actives BACE mediated cleavage of Aβ. Again Aβ enter into the mitochondria and induces free radicals that leads to disruptions of ETC, decrease in cytochrome oxidase activity and the inhibition of ATP which finally leads to neuronal damage and cognitive decline in AD.

It is well documented that reduced cytochrome oxidase activity is correlated with an increased defects in mtDNA in AD (Swerdlow et al., 2014). The brain, due to the presence of high lipid content, high oxygen consumption and low antioxidant defenses, remains the most vulnerable organ to oxidative stress. It is also well documented that free radical generation by mitochondrial dysfunction affect both in neurons and astrocytes during AD. There is also generation of free radicals (${\text{O}}_{\text{2}}^{\cdot —}$) produced in mitochondrial ETC complexes I and III and enzymes of the TCA (α-ketoglutarate dehydrogenase) in AD. From inner mitochondrial membranes hydrogen peroxide (H_2_O_2_) and ${\text{O}}_{\text{2}}^{\cdot —}$ is released to the outer side (cytoplasm) which finally causes oxidation of cytoplasmic proteins (Figure 2; Ylikallio and Suomalainen, 2012). Figure 2 has been adopted and the modified from Reddy and Beal (2008).

Various studies have been performed to understand the relationship between Aβ and mitochondria (Rhein et al., 2009; Ren et al., 2011). Study in transgenic mice (tgAPP/PS1) has shown that the soluble form of Aβ peptide causes a reduced mitochondrial membrane potential (MMP) and ATP levels (Ren et al., 2011). Another study on triple transgenic (APP/TAU/PS2) AD mice showed that decreased mitochondrial protein levels include reduction of the MMP and ATP synthesis (Rhein et al., 2009). *In vivo* study showed that intrahippocampal injection of Aβ peptides damaged mitochondria, causes a decreased Ca^2+^ dependent ATPase activity, MMP and an increased Ca^2+^ levels (Calkins et al., 2011). It has also been reported the correlation between Aβ and tau, it is described that both Aβ and tau work synergistically and leads to the impairment of oxidative phosphorylation (Rhein et al., 2009). Due to the accumulation of Aβ peptides in the mitochondrial import channels (TIM23 and TOM40) and mutant APP in AD brain causes mitochondrial dysfunction (Devi et al., 2006). A study on transgenic mice showed that APP (full length segment) binds to the mitochondria in neuronal cells, causes impaired energy metabolism and mitochondrial dysfunction (Anandatheerthavarada et al., 2003). It has been observed that during AD there is decreased genetic expression of oxidative phosphorylation and energy consumption (Chandrasekaran et al., 1996). A study on triple transgenic mouse (APP/PS1/Tau) showed dysregulated oxidative phosphorylation, glycolysis, TCA cycle, pyruvate metabolism, and mitochondrial protein synthetic pathways by the help of mitochondrial proteome analysis (Chou et al., 2011). Similarly, a study in Aβ transgenic mouse model of AD showed an impaired mitochondrial functions, ROS production, MMP and cytochrome c oxidase (COX) activity (Dragicevic et al., 2010).

## Mitochondrial Therapeutics in Neurodegenerative Diseases

**FIGURE 3:**
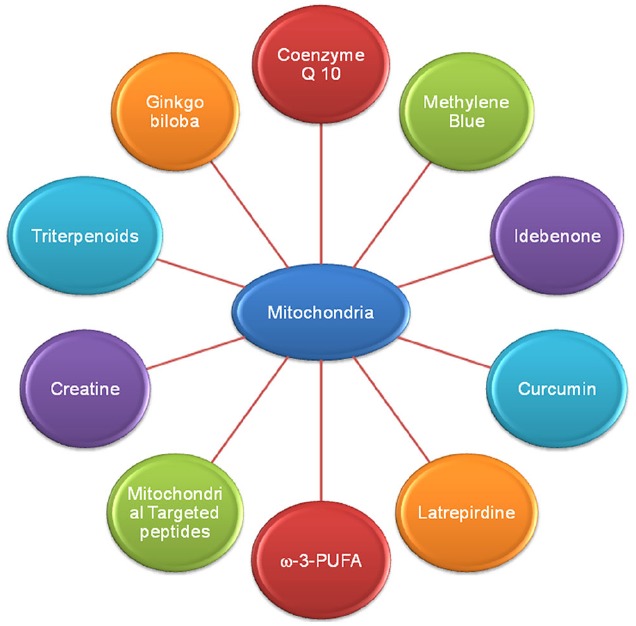
**Various drugs and natural compounds with common potential therapeutic target mitochondria in Alzheimer’s disease**.

As previously discussed, alteration in mitochondrial bioenergetic defects, mitochondrial dynamics and mitochondrial trafficking and oxidative stress ROS mediated mitochondria damage plays a key role in AD, PD, HD, and ALS pathogenesis (Reddy and Beal, 2008; Chaturvedi and Beal, 2013). So, strategies that target mitochondrial dysfunction or the agents which enhance mitochondrial bioenergetics and reversed oxidative stress are potentially needed as therapeutics in AD, PD, HD, and ALS. Here we discussed the therapeutic potential of various bioenergetics and antioxidants (Figure 3) which are either have been used or being in different phases of clinical trials.

### Coenzyme Q 10

#### Mechanism of Action

It is an important cofactor of the ETC, known as ubiquinone. It functions as an electron acceptor for complex I and II and also act as an antioxidant in mitochondria and its membranes (Hargreaves, 2014). It is present in the inner mitochondrial membrane as a cofactor for three mitochondrial complexes (complexes I, II, and III) which play major role in OXPHOS (Hargreaves, 2014). According to Muller, one of the major functions of CoQ10, non-protein component of ETC, is to move and transfer electrons between flavoproteins and cytochromes (Müller et al., 2003). Further, it has been proposed that electrons of ETC must first be interact with CoQ10, a central rate-limiting step, hence considered as major component of ETC (Cleren et al., 2008). The major role of CoQ10 is ATP production. It also possesses antiapoptotic activity by inhibiting activation of the mitochondrial permeability independent of its free radical scavenging property (Matthews et al., 1998). It also acts as a cofactor as well as obligatory cofactor of mitochondrial uncoupling proteins whose activation reduces free radical generation from mitochondrial (Beal and Matthews, 1997; Kašparová et al., 2006).

#### Preclinical Studies

*In vitro* and *in vivo* analysis have suggested the neuroprotective potentials of CoQ10 in AD (Ishrat et al., 2006; Choi et al., 2012). It is a lipophilic anti-oxidant compound that improves cognitive functions, facilitates ATP synthesis and up-regulates mitochondrial function (Turunen et al., 2004). It is well documented that CoQ10 supplementation significantly increases endogenous brain CoQ10 content and provides protection from free radicals mediated oxidative damage biomolecules (Hargreaves, 2014). It also acts as cofactor of dehydrogenase in the ATP production and electrons and proton transport. Previous study on primary neuron culture suggests that CoQ10 significantly inhibit chemically (like paraquat and rotenone) induced mitochondrial dysfunction, maintained MMP, inhibit the mitochondrial ROS generation and neurodegeneration (Chaturvedi and Beal, 2008). CoQ10 also protects cultured cerebellar neurons against excitotoxin induced degeneration. A study on ICV-STZ infused rat showed that CoQ10 supplementation significantly restored choline acetyl transferase activity (Ishrat et al., 2006).

#### Clinical Studies

It is further evident from clinical studies that high doses of CoQ10 are beneficial for the treatment of neurodegenerative diseases (Crane, 2007; Galpern and Cudkowicz, 2007; Lenaz et al., 2007). Clinical study for the evaluation of safety and tolerability of CoQ10 and its combination treatment of mild to moderate AD patients (NCT00117403) is currently in ongoing phase (Müller et al., 2003). In this study, seventy five subjects received CoQ10 2400 mg daily; vitamin E 2400 IU, vitamin C 600 mg and α-lipoic acid 1800 mg; or placebo for a period of 4 months. Various parameters like safety, tolerability, CSF biomarkers of oxidative stress as well as CSF concentration of Aβ (1–40) and (1–42) were evaluated (Müller et al., 2003). Another study, phase-I pilot trial, an open label trial in 15 PD patients, was conducted for the safety and CoQ10 tolerability (Shults et al., 1998). In this study, CoQ10 at different doses 400, 600, and 800 mg/day for a period of 1 month showed significant safety and tolerability profiles along with dose-dependent increase in plasma CoQ10 levels (Shults et al., 1998). Another clinical trial (open label placebo controlled) in ALS patients showed that COQ10 (3000 mg/day) is safe and well tolerated (Ferrante et al., 2005). Recent clinical trial (multicenter, two-stage) in Phase II on CoQ10 showed no significant results between CoQ10 and placebo (Kaufmann et al., 2009).

### Creatine

#### Mechanism of Action

Creatine, a nitrogenous guanidino compound, provides energy to the nerve and muscle cells due to its high energy requirement (Beal, 2011). It is a potent antioxidant and acts as an effective mPTP opening inhibitor and mitochondrial iron accumulation (Beal, 2011). It is present in the form of free creatine and phosphocreatine (PCr) in the human body. It is further gets transformed into PCr by the help of creatine kinase (CK) in skeletal muscle and brain. CK maintains cellular homeostasis by creating a pool of PCr for ATP generation (Adhihetty and Beal, 2008).

#### Preclinical Studies

It exert neuroprotective potential in different neurodegenerative disorders like AD, PD, HD, and ALS (Adhihetty and Beal, 2008; Beal, 2011). *In vitro* studies on neuronal cells have shown that it protects against neurotoxicity induced by 3-nitropropionic acid (3-NP), 1-methyl-4-phenylpyridinium (MPP^+^), and 6-hydroxydopamine (6-OHDA), glucose and serum deprivation (Chaturvedi and Beal, 2008). Creatine administration also known to inhibit the degeneration of dopaminergic neurons and reduced depletion of dopamine levels in PD (Matthews et al., 1999). Besides, creatine supplementation has been proved to be effective and protective against neuronal death caused by NMDA, malonate, Aβ and ibotenic acid induced neurotoxic injuries (Chaturvedi and Beal, 2008). Study on transgenic G93A ALS mice showed that creatine treatment protects motor neurons, brain atrophy and reduced mitochondrial dysfunction (Klivenyi et al., 1999). Besides, creatine in combination with the other bioenergetics compound CoQ10 produces a neuroprotective effects in neurodegenerative diseases (Yang et al., 2009b). Taken together, these studies may suggest creatine, a promising therapeutic agent for various neurodegenerative disorders including AD.

#### Clinical Studies

A clinical study (randomized, double-blind) of creatine in patients with mitochondrial cytopathies, showed constructive effects of creatine (Rodriguez et al., 2007). Another pilot study using creatine treatment showed advantageous effects on the mood swings of PD patients (Bender et al., 2006). In this study creatine (4 g/day) was safe and well tolerated in aged PD patients (Bender et al., 2008). A clinical study (randomized, double-blind) in phase II, on HD patients, showed that creatine (8 g/day) for 16 weeks was found to be safe and well tolerated, and decreased serum 8-hydroxy-2-deoxyguanosine which is the neuropathological marker for oxidative stress (Hersch et al., 2006). Taken together, these clinical studies may suggest a promising, beneficial and neuroprotective role of creatine in neurodegenerative diseases.

### Idebenone

#### Mechanism of Action

It is an analog of CoQ10 consists of short chains of isoprene units, also known as ubiquinone. It has been reported that Idebenone crosses blood brain barrier easily and is well tolerated in humans (Senin et al., 1992). It is also known to possess good anti-oxidant properties (Senin et al., 1992). It belongs to the quinone family and is structurally similar to CoQ10 (Senin et al., 1992; Weyer et al., 1997; Gutzmann and Hadler, 1998).

#### Preclinical Studies

It has been reported that Idebenone produced neuroprotection against Aβ induced neurotoxicity both *in vitro* and *in vivo* (Weyer et al., 1997).

#### Clinical Studies

Clinical study of Idebenone showed its neuroprotective effects in AD patients on Alzheimer’s Disease Assessment Scale (ADAS) score (Gutzmann and Hadler, 1998). It has been proposed that Idebenone (360 mg/day) treatment was safe and well tolerated in AD patients (Gutzmann et al., 2002).

### Latrepirdine

#### Mechanism of Action

Latrepirdine previously known as Dimebon (or Dimebolin), is a non-selective antihistamine (Sachdeva and Burns, 2011). Latrepirdine inhibits weakly acetylcholinesterase and butyrylcholinesterase (Bachurin et al., 2001). Latrepirdine also has known as inhibitor of NMDA receptor and voltage-gated calcium channels. It also possesses the neuroprotective effect mainly by maintaining mitochondrial structure and function (Sachdeva and Burns, 2011). Latrepirdine under both stress and non-stress conditions enhances mitochondrial function (Bachurin et al., 2001; Sachdeva and Burns, 2011). Latrepirdine has also been reported to inhibit Aβ-induced activation of the mPTP which can lead to apoptosis and hence protect neuronal mitochondria from Aβ-induced toxicity (Bachurin et al., 2001). Latrepirdine other functions include an increase in MMP and ATP production (Bachurin et al., 2001; Sachdeva and Burns, 2011).

#### Preclinical Studies

Latrepirdine has been shown to be effective in cognition in animal studies on young/adult mice or rats (Kieburtz et al., 2010; Vignisse et al., 2011). Latrepirdine also known to improve impaired mitochondrial function in AD (Reddy, 2009; Leuner et al., 2012). Latrepirdine (25 μmol/l) has been reported to be protective against the Aβ induced mitochondrial dysfunctions (Reddy, 2009; Leuner et al., 2012). A study on human SH-SY5Y neuroblastoma cells and primary rat cortical neurons showed that latrepirdine (0.1–10 nmol/l) improve mitochondrial function, such as MMP, ATP production and apoptosis (Zhang et al., 2009).

#### Clinical Studies

Phase 2 randomized controlled trial showed that, latrepirdine was well tolerated, safe and significantly enhanced the clinical outcomes in patients suffering from mild-to-moderate AD (Reddy and Reddy, 2011).

### Triterpenoids

#### Mechanism of Action

Triterpenoids are derivatives of oleanolic acid and known to inhibit oxidative stress. They also possess anti-inflammatory properties via inhibition of inflammatory processes; by activation of antioxidant response element (ARE)-Nrf2-Keap1 signaling pathway (Liby et al., 2005). Activation of this pathway leads to the dissociation of Nrf2 from Keap1, translocation to the nucleus and then binding to the ARE promoter sequences which further causes induction of antioxidant and anti-inflammatory genes (Yates et al., 2007). It has recently reported that synthetic triterpenoids (CDDO) causes transcriptional activation of Nrf2, NQO-1, HO-1, glutathione transferase and other cytoprotective enzymes (Liby et al., 2005; Yates et al., 2007). CDDO-methyl amide (2-cyano-N-methyl-3,12-dioxooleana-1,9 (11)-dien-28 amide; CDDO-MA), synthetic triterpenoid, has been discovered as 200,000 times more potent inducer of NQO-1 or suppressor of iNOS than naturally occurring oleanolic acid (Yang et al., 2009a). Further study on same compound CDDO-MA showed that it is a potent and selective activator of the Nrf2/ARE pathway which is neuroprotective in nature (Kaidery et al., 2013).

#### Preclinical Studies

Experimental studies on 3-NP rat model and 1-methyl-4 phenyl-1,2,3,6-tetrahydropyridine (MPTP) mouse model showed that synthetic triterpenoid CDDO-MA, exerts significant neuroprotective effects by potently activating Nrf2/ ARE signaling pathway (Yang et al., 2009c; Kaidery et al., 2013). CDDO-MA has also been reported to inhibit ROS generation, MPTP induced neurodegeneration and dopamine depletion and 3-NP induced striatal lesions (Yang et al., 2009c). It has also been observed that triterpenoids improve the behavioral parameters and survival in transgenic mouse models of variety of neurodegenerative diseases including AD, HD and ALS (Neymotin et al., 2011). These studies may suggest that targeting neuroprotective pathways (Nrf2/ARE) through synthetic triterpenoids (CDDO-MA) could be used as a better therapeutic approach in the treatment and management of neurodegenerative disorders (Dumont et al., 2009; Stack et al., 2010).

### MitoQ

#### Mechanism of Action

MitoQ is formed by coenzyme Q or ubiquinone which is linked to triphosphonium ions via covalent bonding to form mitoquinone (Murphy and Smith, 2007). It is the most widely used antioxidant to target mitochondria. MitoQ demonstrated neuroprotection due to its direct effect on scavenging peroxynitrite, and superoxide, and thus protect mitochondria against lipid peroxidation (Manczak et al., 2010; Calkins et al., 2011; Jin et al., 2014).

#### Preclinical Studies

Studies on both *in vitro* and *in vivo* models showed that it exerts neuroprotective effects in various experimental models (Murphy and Smith, 2007; Jin et al., 2014). It also inhibits mitochondrial fission protein and translocation of pro-apoptotic protein (Bax) in the mitochondria in cellular models of PD (Jin et al., 2014). Although its efficacy in neurodegenerative diseases conditions needs to be further explored and understood.

#### Clinical Studies

A clinical study (double blind), in 128 newly diagnosed untreated patients with PD, MitoQ for 12 months with two doses did not produce any significant improvement according to United Parkinson Disease Rating Scale and PD progression when compared with the placebo control (Snow et al., 2010).

### MitoVitE

#### Mechanism of Action

MitoVitE is also known as Mito tocopherol. Structurally, it is triphenylphosphonium (TPP) conjugated to α-tocopherol moiety of vitamin E via two-carbon chain. It also protects mitochondria from oxidative stress via inhibition of lipid peroxidation (Smith et al., 1999).

#### Preclinical Studies

*In vivo* study, showed that intravenous injection of MitoVitE accumulated rapidly in the heart, brain, muscle, liver, and kidney tissues which are mostly affected by mitochondrial dysfunction and oxidative stress (Smith et al., 2003). An *in vitro* cellular model also demonstrated its efficacy against mitochondrial oxidative stress, reduction of peroxide mediated oxidative stress, peroxide-induced caspase activation and oxidative stress-induced cell death (Jauslin et al., 2003; Dhanasekaran et al., 2004; Hughes et al., 2005). But till date, its efficacy and therapeutic potential in PD patients has not been investigated.

### MitoTEMPOL

#### Mechanism of Action

It is another TPP^+^ derivative which consists of stable piperidine nitroxide radical TEMPOL (4-hydroxy-2,2,6,6,-tetramethylpiperidine- 1-oxyl). The most important property is to accept an electron from hydroxylamine (potent radical scavenger). It also acts as a SOD mimetic, whose function is to converts superoxide molecules into water to detoxify ferrous iron into ferric iron.

#### Preclinical Studies

*In vitro* study has shown its beneficial effects against mitochondrial dysfunction and mitochondria mediated oxidative stress (Trnka et al., 2008). However, its neuroprotective potential in different neurological problem is yet to be explored and understood.

### SS (Szeto-Schiller) Peptides

#### Mechanism of Action

They are mitochondrial targeted peptides; act as novel anti-oxidants, helpful in restoring mitochondrial functions (Szeto, 2008). A study in isolated mitochondria showed that these peptides decreases mitochondrial ROS generation; inhibit mitochondrial swelling and cytochrome c release (Szeto, 2008; Manczak et al., 2010; Calkins et al., 2011). Further, the modification in their structure via addition of a tyrosine or modified tyrosine moiety causes improvement in their free radical scavenging properties, and; apoptosis (Manczak et al., 2010; Calkins et al., 2011; Smith and Murphy, 2011). Besides, their related peptides (SS-31 and SS-20) have also been shown to restore mitochondrial functional properties by reducing inhibition of the mitochondrial ETC, apoptosis and oxidative stress (Smith and Murphy, 2011).

#### Preclinical Studies

A study showed that SS peptides decrease mitochondrial ROS generation, inhibit mitochondrial swelling, and reduce cytochrome c release from mitochondria in different experimental models (Szeto, 2008). These findings have suggested their therapeutic potential in neurodegenerative disorders including AD.

### Methylene Blue

#### Mechanism of Action

Methylene blue (MB) is an FDA-approved drug used for the treatment of various diseases like malaria and some psychiatric disorder for more than 100 years (Naylor et al., 1988; de-Oliveira and Guimarães, 1999; de Oliveira et al., 2000; Oz et al., 2012). MB possess better pharmacokinetic profile, readily absorbed in blood and quickly distributed to various organs (Peter et al., 2000; Rengelshausen et al., 2004). Other functions of MB includes cognition enhancing properties and increase oxygen consumption efficacy in isolated mitochondria (Zhang et al., 2006). MB (1 mg/kg) has also reported to increase COX activity and thereby improving energy functions in AD brains (Gonzalez-Lima et al., 1997; Valla et al., 2001; Callaway et al., 2004; Hauptmann et al., 2009).

#### Preclinical Studies

It is now well known that complex I of ETC is the most susceptible to oxidative stress in AD (Leuner et al., 2007). A study on mice showed that MB (0.07 mg/kg) when administered in the eyes of mice (intravitreally) by the help of microinjector significantly reverses rotenone induced mitochondrial dysfunction (Complex I inhibitors; Zhang et al., 2006). Neuroprotective effect of MB (0.15–4.0 mg/kg b.w, i.p.) has been well demonstrated in animal models of cognitive dysfunction (Deiana et al., 2009). It has also been reported that disturbances in the ETC generally elevate ROS level, which in turn increases Aβ production, Aβ impairs mitochondrial function, and hence finally a vicious cycle is initiated (Leuner et al., 2012). MB (as a redox compound), prevents the reduction of molecular oxygen to superoxide (Leuner et al., 2007). Thus MB acts as both mitochondrial restorer as well as antioxidant.

### Ginkgo biloba

#### Mechanism of Action

EGb761^®^ is a standardized extracts of *Ginkgo biloba*, herbal drug, used for the improvement of cognitive dysfunction. The extract consists of 24% flavonoids and 6% terpenes (Bedir et al., 2002; Sastre et al., 2002; Abdel-Kader et al., 2007).

#### Preclinical Studies

Study on PC12 cells showed that EGb761^®^ significantly improved MMP and restored ATP levels after sodium nitroprusside (a nitric oxide donor) induced mitochondrial damage (Abdel-Kader et al., 2007). Similar results have been observed in isolated brain cells and brain mitochondria after treatment with EGb761^®^ (Eckert et al., 2005). *In vitro* studies on PC12 cells having mutant APP, showed that EGb761^®^ (0.01 mg/ml) treatment leads to enhanced Aβ production and increase mitochondrial functions (Eckert et al., 2005; Abdel-Kader et al., 2007). Animal study involving EGb761^®^ (100 mg/kg) treatment for 14 days significantly improved complexes I, IV, and V activities of the mitochondria and alleviated nitrosative stress (Abdel-Kader et al., 2007). Further, protective mechanism is due to the presence of terpene lactones that restore mitochondrial functional properties and acts as a free radical scavenger (Abdel-Kader et al., 2007). Recently EGb761^®^ showed an improvement in neuroplasticity, oxidative stress, long-term potentiation, spine density, neurogenesis (Müller et al., 2012). Besides, EGb761^®^ alters APP processing, by upregulating the activity of α-secretase in hippocampal region (Colciaghi et al., 2004).

#### Clinical Studies

Clinically *Ginkgo biloba* has been well studied in dementia due to mitochondrial restoring properties (Stoll et al., 1996; Tang et al., 2002; Abdel-Kader et al., 2007; Wang et al., 2010; Weinmann et al., 2010). However, its neuroprotective mechanism is still not clear.

### Curcumin

#### Mechanism of Action

Also known as diferuloylmethane, yellow pigment derived from the rhizome part of the turmeric plant (*Curcuma longa*, Sikora et al., 2010). It is lipophilic phenolic diferuloylmethane and possesses varieties of pharmacological functions, including anti-inflammatory, antioxidative, anti-proliferative, cholesterol- lowering, and neuroprotective (Bengmark, 2006; Lapchak, 2011; Vauzour, 2012). Curcumin affects various pathways involved in AD like neuroprotective processing of APP, tau phosphorylation, neuroinflammation, or oxidative stress (Zhu et al., 2004).

#### Preclinical Studies

A preclinical study on PC12 neuronal cells, showed that curcumin (25 μmol/l) given for 2 h maintained redox potential and respiratory functions of mitochondria after hydroxynonenal (4-HNE) treatment (Raza et al., 2008). Another study on neurons of rat cortices treated with tert-butyl hydroperoxide (t-BHP) to induce oxidative stress showed that curcumin (2.5–20 μmol/l) improves MMP and cytochrome c release, inhibits the activation of caspase-3, and altered the expression of Bcl-2 (Zhu et al., 2004). A study on homocystein-induced rat aging model showed that curcumin (5, 15, or 45 mg/kg) treatment significantly decreased MDA and superoxide anion levels and finally improves learning and memory (Ataie et al., 2010). Another study in aged mice showed curcumin’s antioxidative effects with significant decrease in ROS level and protein carbonylation (Dkhar and Sharma, 2010). In another *in vivo study*, chronic administration of d-galactose causes cognitive dysfunction, oxidative stress, and impaired mitochondrial enzyme complex I, II, and III levels and curcumin treatment (15 and 30 mg/kg) for 6 weeks significantly improved cognition, oxidative stress, and restored mitochondrial enzyme complex activity as compared to control (Kumar et al., 2011). Curcumin (120 mg/kg) in a diabetic rat model, up- regulate mitochondrial complex activities and increase ATP level in the brain (Rastogi et al., 2008).

#### Clinical Studies

Curcumin, due to its poor bioavailability and poor water solubility, may have limited clinical trials (Belkacemi et al., 2011). So, for effective therapy, new delivery strategies may need to be developed.

### Omega-3 Polyunsaturated Fatty Acids

#### Mechanism of Action

Omega-3 polyunsaturated fatty acids (ω-3 PUFAs) are groups of essential fatty acids. It functions as energy substrates and is a part of integral membrane components. Therefore, plays major roles in the management of neurological function (Cole et al., 2009). A recent study showed that ω-3 PUFAs exerts neuroprotective role in the cognitive dysfunction (Eckert et al., 2011). Brain fulfills the need of ω-3 PUFAs by the delivery through blood because of their limited synthesis (Cole et al., 2009). It has been reported that decreased levels of ω-3 PUFAs or fish consumption increases risk for age-related cognitive deficits such as AD (Cole et al., 2009).

#### Preclinical Studies

*In vitro* studies in HEK-APP cells showed beneficial effects of ω-3 PUFAs (20 μmol/l) as it significantly increased mitochondrial membrane properties and processing of non-amyloidogenic APP, which causes enhanced secretion of sAPPα, that in turn protect against mitochondrial dysfunction and apoptosis (Eckert et al., 2011). It has also been reported that supplementations with ω-3 PUFAs increase membrane phospholipid docosahexaenoic acid (DHA). ω-3 PUFAs also known to improve the functions of complexes I and IV of mitochondrial respiratory chain, mitochondrial respiration and lipid metabolism (Barceló-Coblijn et al., 2003; Stanley et al., 2012).

### Current Trends in Management of Mitochondrial Related Diseases

#### Mitochondrial Replacement Therapy

It has been reported that each cell contains almost 1,000 to 100, 000 copies of mtDNA which via maternal inheritance transfers to the offspring (Swerdlow et al., 2014). Various techniques have been introduced like genetic screening of embryos, and have been reported to partially decrease the risk of mitochondrial diseases transmitted from mother to offspring being (Tachibana et al., 2009; Reinhardt et al., 2013). Recently a new technique is introduced, mitochondrial replacement therapy, in which mitochondria healthy in nature are taken from a donor, is under investigation. A limitation of this approach is the combination of the genetic material from three different people, which causes array of ethical, safety and medical expostulations (McNamee, 2015).

#### Mitochondrial Editing Technique

It has been reported first time the gene-editing technology, the technique used to prevent mitochondrial diseases of humans from being transferred from female their offspring. Researchers from Salk Institute for Biological Studies in La Jolla, CA, USA discover mitochondrial editing technique for the treatment of various neurodegenerative disorders. It is safe, simple and more ethical as compared to mitochondrial replacement therapy as it does not involve donor DNA. It is an alternative approach which involves editing the mutated DNA of mouse using enzymes called as restriction endonucleases and transcription activator-like effector nucleases (TALENs). According to the author of the Salk Institute for Biological Studies, “this technique is based on a single injection of mRNA into a mother’s oocytes or early embryos and therefore could be easily implemented in IVF (*in vitro* fertilization) clinics throughout the world.” As mutation in mitochondrial DNA is involved in a variety of neurodegenerative disorders, cancer and aging, this technology may have broader potential therapeutic significance for the prevention of transmission of disease-causing mutations from mother to future generations, said by one of the author Belmonte (McNamee, 2015).

## Conclusion

As in neurodegenerative diseases the two pathophysiological hallmarks mitochondrial dysfunction and oxidative stress were involved. In AD, apart from theses two other hallmarks are impaired energy metabolism, excessive ROS generation, impaired Ca^2+^ signaling, and increased mtDNA mutations and disturbance in OXPHOS. Mitochondrial dysfunction plays critical role in AD and can be considered as an important target for the treatment of clinical symptoms of AD. Previous evidences have shown the potential efficacy of various bioenergetics and antioxidants having in the treatment of AD, for example coenzyme Q10, carnitine, α-lipoic acid, Idebenone, mito-targeted compounds like Mito Q, Mito Vit E and Mito TEMPOL, *Ginkgo biloba*, curcumin and omega-3 polyunsaturated fatty acids. Preclinical data of the above mentioned drugs indicated beneficial effects, whereas most of the clinical trials did not. This might be because mitochondrial dysfunction represents an early event in AD progression and pharmacological interventions might came in the later event in AD. Targeting abnormal mitochondrial dynamics in AD may represent a novel therapeutic strategy which leads to the development of new mitochondrial targeted bioenergetics and antioxidants. Mitochondrial dysfunction and oxidative stress are also useful in defining the complex pathophysiology of this disabling disease. Still, more studies are urgently required to focus on the therapeutic interventions before the disease progresses.

### Conflict of Interest Statement

The authors declare that the research was conducted in the absence of any commercial or financial relationships that could be construed as a potential conflict of interest.
